# Rotavirus A strains obtained from children with acute gastroenteritis in Mozambique, 2012-2013: G and P genotypes and phylogenetic analysis of VP7 and partial VP4 genes

**DOI:** 10.1007/s00705-017-3575-y

**Published:** 2017-10-20

**Authors:** Eva Dora João, Amy Strydom, Hester G. O’Neill, Assa Cuamba, Marta Cassocera, Sozinho Acácio, Inácio Mandomando, Lithabiso Motanyane, Nicola Page, Nilsa de Deus

**Affiliations:** 1 Centro de Investigação em Saúde de Manhiça (CISM), Manhiça, Mozambique; 2 Institute of Hygiene and Tropical Medicine, Lisbon, Portugal; 3 Department of Microbial, Biochemical and Food Biotechnology, University of the Free State, Bloemfontein, South Africa; 4 Faculdade de Medicina, Universidade Eduardo Mondlane, Maputo, Mozambique; 5 Instituto Nacional de Saúde, Maputo, Mozambique; 6 Centre for Enteric Diseases, National Institute for Communicable Disease (NICD), A division of National Health Laboratory Services, Johannesburg, South Africa; 7 Department of Medical Virology, Faculty of Health Sciences, University of Pretoria, Pretoria, South Africa

## Abstract

In Mozambique rotavirus (RV) was shown to be the greatest cause of acute diarrhoea in infants from 0 to 11 months, and in 2015, national rotavirus vaccination was introduced. As with other developing countries, there is very limited active strain characterisation. Rotavirus positive clinical specimens, collected between 2012 and 2013, have now provided information on the genotypes circulating in southern Mozambique prior to vaccine introduction. Genotypes G2 (32.4%), G12 (28.0%), P[4] (41.4%) and P[6] (22.9%) (n = 157) strains were commonly detected with G2P[4] (42.3%) RVs being predominant, specifically during 2013. Phylogenetic evaluation of the VP7 and VP8* encoding genes showed, for the majority of the Mozambican strains, that they clustered with other African strains based on genotype. RVA/Human-wt/MOZ/0153/2013/G2P[4], RVA/Human-wt/MOZ/0308/2012/G2P[4] and RVA/Human-wt/MOZ/0288/2012/G12P[8] formed separate clusters from the other Mozambican strains with similar genotypes, suggesting possible reassortment. Amino acid substitutions in selected epitope regions also supported phylogenetic clustering. As expected, the VP7 and VP8* genes from the Mozambican strains differed from both the RotaTeq^®^ (SC2-9) G2P[5] and Rotarix^®^ (A41CB052A) G1P[8] genes. This study provides information on the genetic diversity of rotavirus strains prior to vaccine introduction and generates baseline data for future monitoring of any changes in rotavirus strains in response to vaccine pressure.

**Electronic supplementary material:**

The online version of this article (doi:10.1007/s00705-017-3575-y) contains supplementary material, which is available to authorized users.

## Introduction

Rotaviruses are one of the leading causes of severe-dehydrating diarrhoea in infants and young children. The number of global deaths due to rotavirus infection in children under the age of five was estimated to be 215 000 in 2013; of these deaths, 56% occurred in Sub-Saharan Africa [1]. Rotaviruses are taxonomically classified within a genus of the *Reoviridae* family and contain an 11-segment double-stranded RNA (dsRNA) genome. The dsRNA segments encode six structural (VP1–VP4, VP6 and VP7) and six non-structural (NSP1–NSP6) proteins. The structural viral proteins (VPs) are assembled in three concentric layers enclosing the genomic segments, the viral RNA-dependent RNA polymerase (VP1) and the viral capping enzyme (VP3). The three capsid layers consist of 60 dimers of the inner capsid protein, VP2, 260 trimers of the middle layer protein, VP6, and 780 monomers of the glycosylated VP7 protein. Spike proteins formed by 60 trimers of the protease-sensitive VP4 protein protrude on the surface of the virion [2].

Rotaviruses are classified into several groups (A – H) based on serotyping and/or genotyping of VP6 [3]. Recently, strains for rotavirus group I and group J have also been proposed [4, 5]. A dual typing system, based on the genome segments encoding the VP4 (P genotypes) and VP7 (G genotypes) proteins, is commonly used in surveillance studies of type A rotaviruses. Thus far, 37 P types and 27 G types have been described globally [6, 7] but another 13 P and 7 G types have been proposed, as of April 2017 (https://rega.kuleuven.be/cev/viralmetagenomics/virus-classification). The most prevalent rotavirus A strains found in humans are the G1, G2, G3, G4, G9 and G12 genotypes in combination with P[4], P[6] and P[8] [8, 9]. Unlike infections in developed countries, where G1P[8] strains cause almost 70% of the rotavirus infections, wide strain diversity is associated with infections in African countries [10].

Mozambique has a high diarrhoeal disease burden with more than 13 000 deaths occurring annually in children under 5 years of age [11]. The country participated in the global enteric multi-centre study (GEMS) to establish the burden of diarrhoea and disease aetiologies in sub-Saharan Africa and Asian countries. Results from the GEMS study showed that in Mozambique rotavirus group A (RVA) was the most significant cause of acute diarrhoea in infants from 0 to 11 months [12]. However, no genetic characterization of the rotavirus strains was performed during this study.

The first description of RVA G- and P-genotypes in Mozambique was recently published, describing strains circulating in Chókwè, southern Mozambique in 2011 [13]. Between 2012 and 2013, an epidemiological study in children under 5 years of age in both an urban area (Mavalane, Maputo) and a rural district (Manhiça) was initiated, with RVA detected in 42.4% (163/384) of hospitalized diarrhoea cases [14]. Here we report the G- and P-genotypes of the RVA strains detected in the study and describe the molecular epidemiology of the VP7 and VP8* encoding genes of these selected strains. Since vaccination against RVA was introduced in Mozambique in 2015, the study provides baseline data on pre-vaccination RVA strains circulating in Mozambique.

## Materials and methods

### Rotavirus strains

Between February 2012 and September 2013, a cross-sectional study was conducted at two sites in Mozambique; Mavalane General Hospital (MGH) in Maputo and Manhiça District Hospital (MDH) in the Manhiça district (Supplementary Material 1). Faecal samples were collected from children under-five-years old that had been admitted and hospitalized with acute diarrhoea (duration no more than 7 days); defined by three or more looser-than-normal stool passages or watery diarrhoea in the 24 hours prior to the hospital visit. A total of 163 rotavirus positive specimens were detected by EIA (Oxoid, UK) and kept for further analysis [14].

### RNA extraction and RT-PCR genotyping

RNA extraction was performed from 10% faecal sample suspensions in distilled water using the QIAamp Viral RNA Kit (Qiagen, USA), following the manufacturer’s instructions. Total RNA was eluted in 60 µL AVE buffer. The extracted RNA was amplified in a reverse-transcription polymerase chain reaction (RT-PCR) with AMV reverse transcriptase (Promega, USA) and Taq DNA polymerase (Promega, USA). The reactions targeted the full VP7 encoding gene (sBeg9/End9; 1062 bp) and the partial VP4 encoding gene for amplification (VP8*; Con3/Con2; 876 bp) [15, 16] as described previously.

G genotyping was carried out by semi-nested, type-specific, multiplex PCR as described previously [15, 17]. Amplicon products from the first round RT-PCR were added to a second round multiplex PCR containing primer RVG9, as well as primers aBT1, aCT2, mG3, aDT4, aAT8v, mG9, G10, and G12b, specific for G types 1, 2, 3, 4, 8, 9, 10, and 12, respectively.

A similar approach was followed for P genotyping. First round amplification with primers Con2 and Con3 was added to a reaction containing Con3 and primers 1T-1D, 2T-1, 3T-1, 4T-1, 5T-1, mP11 and p4943, which are specific for types P[8], P[4], P[6], P[9], P[10], P[11] and P[14] respectively [16, 18]. The amplicon size generated in the genotyping reactions denotes the respective G and P type and was determined by analysis on a 1.5% agarose gel, as specified previously [15–18].

### Nucleotide sequencing

Nucleotide sequencing of selected VP7 and VP8* encoding genes was performed using the dideoxynucleotide chain termination method (Supplementary Material 2). Specifically, AMV reverse transcriptase (Thermo Scientific) and KAPA HiFi polymerase (Kapa Biosystems) were used in RT-PCR reactions to amplify the VP7 (sBEG/End9) [15] and VP8* (Con3/Con2) [16] encoding genes. PCR amplicons were purified using the NucleoSpin® PCR clean-up and Gel extraction kit (Macherey-Nagel, Germany), according to the manufacturer’s instructions. The nucleotide sequences of these amplicons were determined using the same forward and reverse primers used for amplicon generation and the BigDye terminator v.3.1 kit (Applied Biosystems), again according to the manufacturer’s instructions. Sequencing products were analysed on an ABI 3130 Genetic Analyzer (Applied Biosystems) and the resulting electropherograms were edited in CLC Bio (Qiagen).

### Data analyses

Sequences were analysed using the Nucleotide Basic Local Alignment Search Tool (BLASTn) and genotypes were confirmed with the online database Virus Pathogen Database and Analysis Resource (ViPR) [19]. The nucleotide sequences of the Mozambican RVA strains have been submitted to GenBank with accession numbers KY315699-KY315722 being assigned to the VP8* and KY426071-KY426094 to the VP7 encoding sequences, respectively. Phylogenetic analyses were carried out using MEGA 7.0.14 [20]. A MUSCLE alignment was performed to align the Mozambican sequences with relevant nucleotide sequences obtained from GenBank. Phylogenetic trees were built with the Maximum Likelihood method using 1000 bootstraps. The Tamura 3 correction parameter was determined as the best substitution model [21] for the VP7 encoding sequences and the Hasegawa-Kishino-Yano model [22] for the VP8* encoding sequences. Amino acid sequences were aligned in Clustal Omega and epitopes were identified as described by Aoki and co-workers [23]. Amino acid sequences of the VP7 Mozambican strains were compared to those of the vaccine strains, RotaTeq^®^ (SC2-9) G2P[5] (VP7: GU565068) and Rotarix^®^ (A41CB052A) G1P[8] (VP7: JN849114) whereas VP8* Mozambican sequences were compared to RotaTeq^®^ (WI79-4) G6P[8] (VP4: GU565044) and Rotarix^®^ (A41CB052A) G1P[8] (VP4: JN849113).

## Results

### G- and P-genotypes

**Fig. 1:**
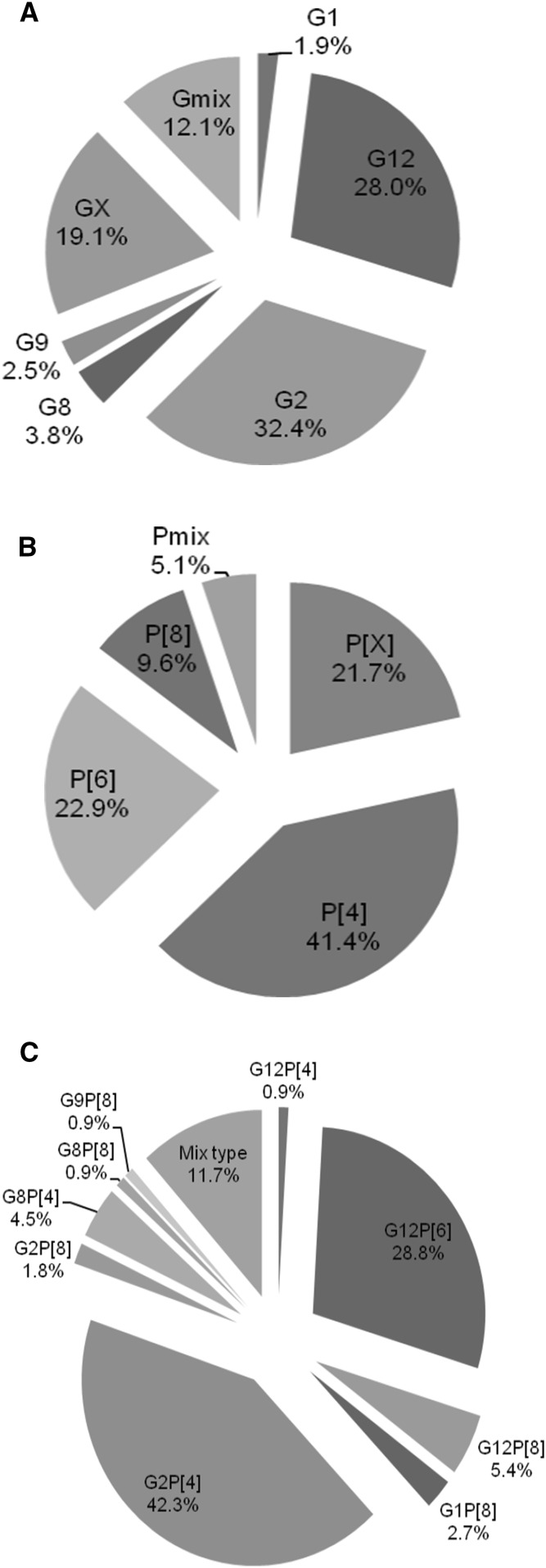
Frequency of partial G (**A**), P (**B**) and G/P (**C**) types determined in rotavirus positive samples. Samples were taken between February 2012 and September 2013 from children under 5 years of age with acute diarrhoea, in both Manhiça District Hospital and Mavalane General Hospital, Mozambique. A: G partially typed samples. B: P Partially typed samples. C: G/P fully typed samples. X refers to strains that were non-typeable for G or P. Gmix, Pmix and mix type represent samples with more than one G or P type

Typing could be carried out for 157 of the 163 RVA ELISA-positive samples. The G and P genotypes for 70.7% (111/157) of the specimens could be determined; while 8.3% (13/157) were partially typed for the G genotype and 10.2% (16/157) for the P genotype. For 17 specimens (10.8%), neither the G nor the P genotype could be determined. The most common G genotype was G2, (32.4%; 51/157), followed by G12 (28.0%; 44/157) and, mixed types (12.1%; 19/157), including G9G2, G12G9 and G12G8 (Fig. 1A). Genotype G8 (3.8%; 6/157), G9 (2.5%; 4/157) and G1 (1.9%; 3/157) were identified at lower frequencies. The most frequently detected P genotypes were P[4] at 41.4% (65/157) and P[6] at 21.7% (36/157; Fig. 1B). The P[8] genotype was determined at a frequency of 9.6% (15/157). A total of 21.7% (34/157) of P genotypes could not be determined.

For fully typed samples (n = 111), 19 different G and P combinations were observed. The most common G/P combinations detected were G2P[4] at a frequency of 42.3% (47/111) and G12P[6] at 28.8% (32/111; Fig. 1C). Other genotypes detected at lower frequencies included G8P[4] (4.5%; 5/111) and G12P[8] (5.4%; 6/111). Seven samples had mixed G genotypes, three samples had mixed P genotypes and three samples had both mixed G and P genotypes (Fig. 1C). Comparison of strain distribution by area revealed that G2P[4] was most prevalent (39.4%, 26/66) in Manhiça (rural area) (Table 1) while in Mavalane (urban area) (Table 1), G12P[6] strains (28.6%, 26/91) predominated. Mavalane also showed higher strain diversity with more mixed genotypes detected.

**Table 1:** Frequency of G/P genotype combinations detected in Manhiça (rural) and Mavalane (urban) area

Genotypes	Study area n (%)	
	Manhiça^1^	Mavalane^2^
G12P[4]	0	1 (1.1)
G12P[6]	6 (9.1)	26 (28.6)
G12P[8]	1 (1.5)	5 (5.5)
G12P[X]	2 (3.0)	1 (1.1)
G1P[8]	1 (1.5)	2 (2.2)
G2P[4]	26 (39.4)	21 (23.1)
G2P[8]	1 (1.5)	1 (1.1)
G2P[X]	0	2 (2.2)
G8P[4]	0	5 (5.5)
G8P[8]	0	1 (1.1)
G9P[8]	0	1 (1.1)
G9P[X]	2 (3.0)	0
GXP[4]	7 (10.6)	2 (2.2)
GXP[6]	1 (1.5)	0
GXP[8]	0	1 (1.1)
^a^Gmix-P[X]	8 (12.1)	1 (1.1)
^b^GX-Pmix	0	2 (2.2)
^c^Gmix-P	0	7 (7.7)
^d^G-Pmix	0	3 (3.3)
^e^Gmix-Pmix	0	3 (3.3)
NT	11 (16.7)	6 (6.6)
Total	66 (100)	91 (100)

X refers to strains that were non-typeable for G or P, NT refers to strains not typed for both G and P. Gmix and Pmix represents samples with more than one G or P type detected

^1^ Manhiça. ^a^Gmix-P[X]: G12G8P[X] (12.1%)

^2^ Mavalane. ^a^Gmix-P[X]: G9G8G2P[X], (1.1%). ^b^GX-Pmix: GXP[6]P[4] (1.1%), GXP[8]P[6] (1.1%). ^c^Gmix-P: G12G8P[4] (2.2%), G12G8P[6] (1.1%), G12G9P[6] (1.1%), G9G2P[4] (1.1%), G9G2P[6] (1.1%), G9G2P[8] (1.1%). ^d^G-Pmix: G9P[8]P[4] (1.1%), G12P[8]P[6] (2.2%). ^e^Gmix-Pmix: G12G8P[6]P[4], (1.1%), G12G9P[8]P[6], (2.2%)

**Fig. 2:**
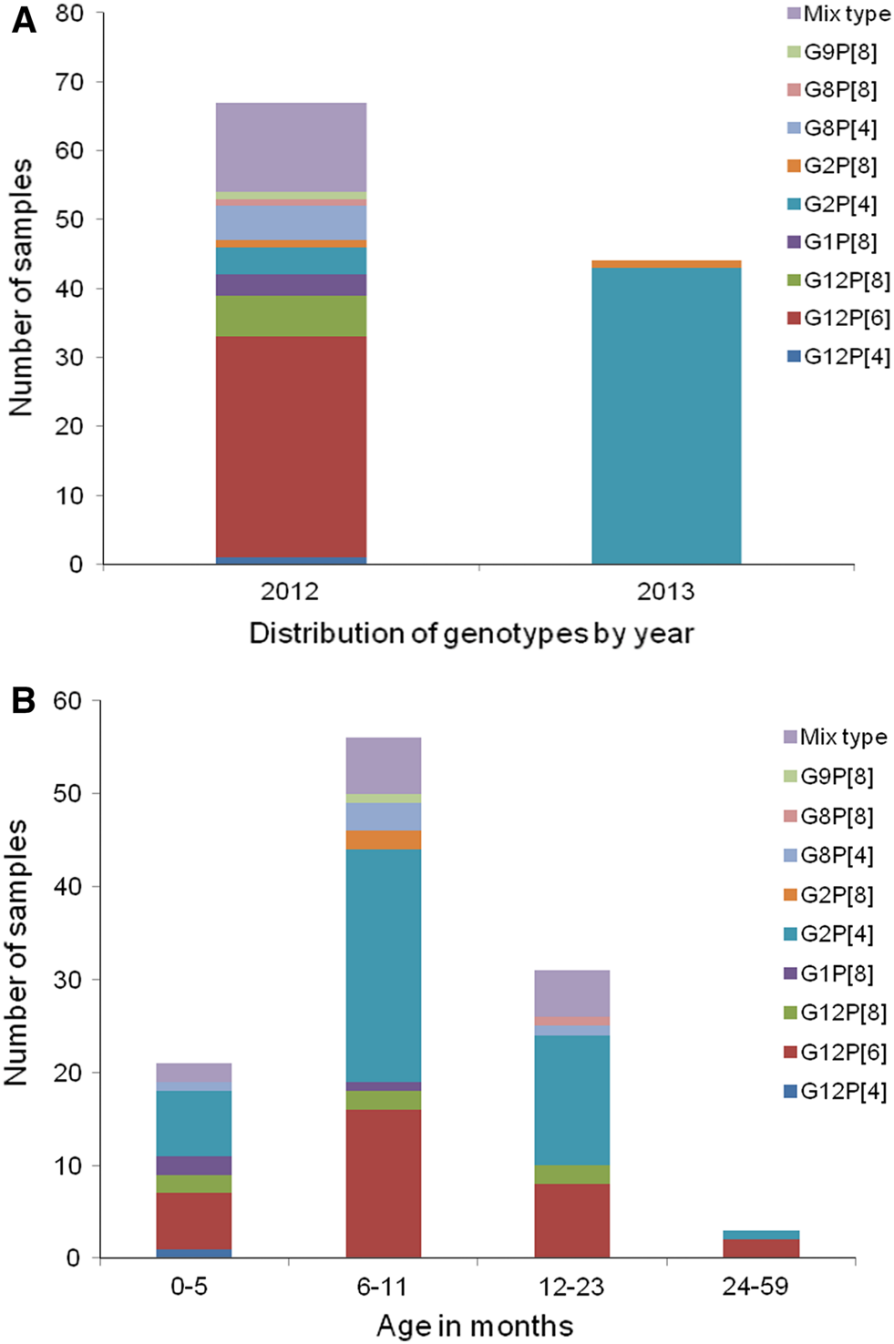
Frequency of G/P genotype combinations detected in rotavirus positive samples from children with acute diarrhoea under 5 years of age; distributed by year of study and age of children. A: Distribution of rotavirus strains by year. B: Distribution of rotavirus strains by age of the children. Mixed type represents samples with more than one G or P type detected

Analyses of the temporal distribution of fully typed strains (n = 111) showed that in 2012 G12P[6] was most prevalent (47.8%; 32/67), followed by mixed infections (19.4%; 13/67; Fig. 2A). In addition, G12 was detected in combination with P[8] (9.0%; 6/67) and G8 in combination with P[4] (7.5%; 5/67). All mixed infections were detected in 2012. In 2013, the predominant strain was G2P[4] (97.7%; 43/44). A low frequency of G2P[8] (2.3%; 1/44) was also detected (Fig. 2A). In addition, greater strain diversity was observed in 2012, when compared to 2013 (Fig. 2A). Analysis of G and P types by age did not indicate any age-specific genotype association (Fig. 2B). In general, genotypes G2P[4] and G12P[6] were frequently observed in all age groups.

A total of 24 G and 23 P genotypes were sequenced and the sequencing results correlated with the genotyping results, apart from the P genotype for sample RVA/Human-wt/MOZ/0042/2012/GXP[6] (Supplementary Material 2). The partial VP4 encoding gene was identified as P[6] by sequencing and as P[8] by genotyping PCR. Five G2 strains (RVA/Human-wt/MOZ/0113/2013/G2P[4], RVA/Human-wt/MOZ/0117/2013/G2P[4], RVA/Human-wt/MOZ/0151/2012/G2P[4], RVA/Human-wt/MOZ/0153/2012/G2P[4] and RVA/Human-wt/MOZ/0412/201/G2P[X]) that could not be typed by PCR were identified by sequencing. Likewise, the P genotype for two samples (RVA/Human-wt/MOZ/0060/2012/GXP[8] and RVA/Human-wt/MOZ/0441/2013/G2P[4]) were elucidated by sequencing and were identified as P[8] and P[4], respectively. Conversely, the G genotyping PCR results for two samples (RVA/Human-wt/MOZ/0060/2012/GXP[8] and RVA/Human-wt/MOZ/0289/2012/GXP[6] could not be confirmed as G12 with nucleotide sequencing. The VP8* encoding gene of RVA/Human-wt/MOZ/0277/2012/G12P[X] could not be confirmed as P[6].

Since the G2 genotype was missing for several samples (by genotyping PCR), the primer binding site of the G2 forward genotyping primer (aCT2) was analysed (Supplementary Material 3). Although differences were observed between the last three nucleotides of the aCT2 primer and the G2 sequences of Mozambican strains, most Mozambican G2 strains could be genotyped using the aCT2 primer (RVA/Human-wt/MOZ/0113/2013/G2P[4], RVA/Human-wt/MOZ/0117/2013/G2P[4], RVA/Human-wt/MOZ/0151/2012/G2P[4], RVA/Human-wt/MOZ/0153/2012/G2P[4] and RVA/Human-wt/MOZ/0412/201/G2P[X]; Supplementary Material 3). The incorrect typing of strain RVA/Human-wt/MOZ/0042/2012/GXP[6] as P[8] was also investigated by comparing the primer binding regions for the respective genotypes (Supplementary Material 3). Again, it was not clear why the P[6] primer (3T-1) failed to detect the P[6] genotype using the genotyping PCR. Fifteen of the 18 base pairs in the P[8] genotyping primer (1T-1D) did not match the sequence of RVA/Human-wt/MOZ/0042/2012/GXP[6] and it is therefore unclear why the RVA/Human-wt/MOZ/0042/2012/GXP[6] strain was incorrectly genotyped as P[8].

### Phylogenetic analysis

**Fig. 3:**
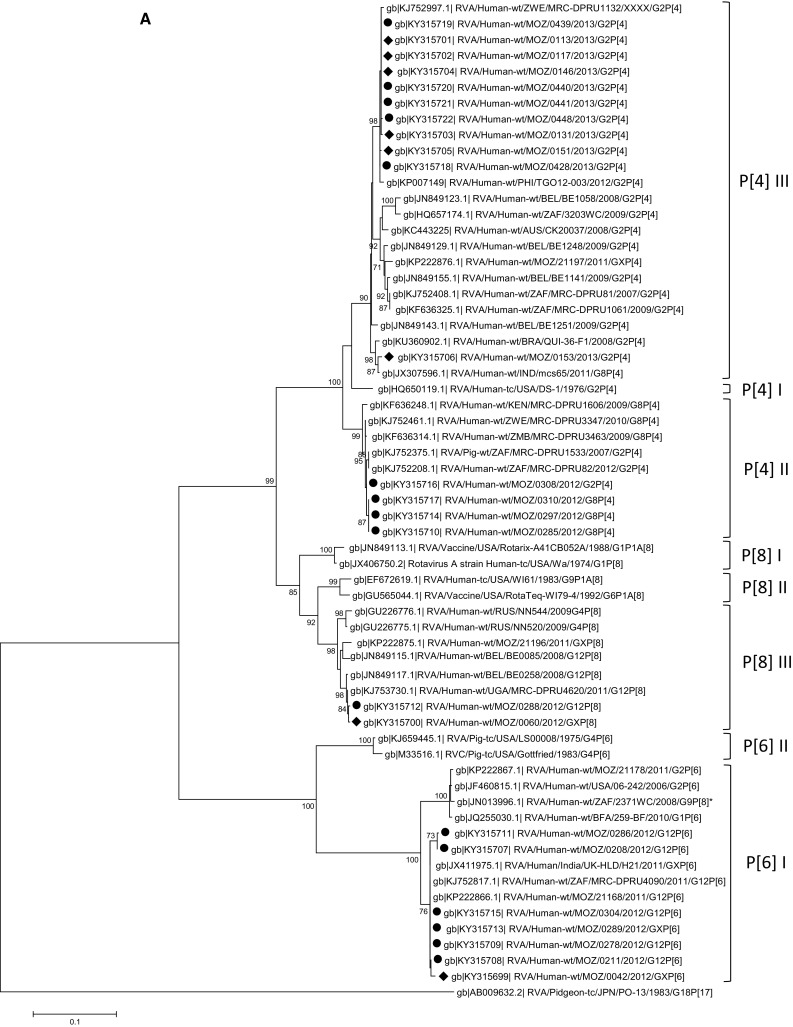

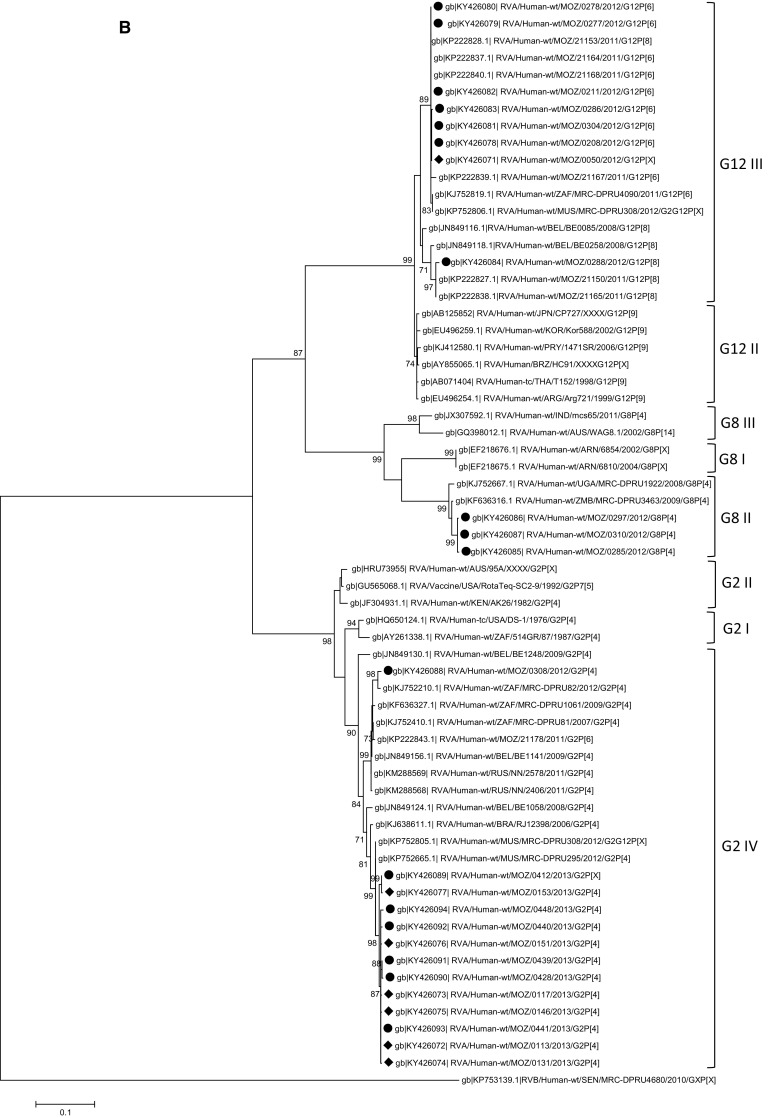
Phylograms based on analyses of nucleotide sequences encoding for RV VP8* (A) and VP7 (B). Mozambican strains from Mavalane are indicated with black ovals (∙) and those from Manhiça are indicated with black diamonds (◆). Accession numbers are included for all strains. The evolutionary history was inferred using the Maximum Likelihood method. The percentage of replicate trees in which the associated taxa clustered together in the bootstrap test (1000 replicates) are shown next to the branches and values < 70 are not shown. The trees are drawn to scale, with branch lengths in the same units as those of the evolutionary distances used to infer the phylogenetic tree. A: Phylogenetic tree of VP8* encoding sequences. The tree is based on the Hasegawa-Kishino-Yano model. *RVA/Human-wt/ZAF/2371WC/2008/G9P[8] detected in South Africa contained multiple genotypes and the sequence in this analysis is based on the P[6] genotype [23]. B: Phylogenetic tree of VP7 encoding sequences. The tree is based on the Tamura 3-parameter

Nucleotide-based phylogenetic trees were built for the VP8* and VP7 encoding genes (Fig. 3A and B). Ten of the 15 Mozambican P[4] strains clustered together with strains from the Philippines and Zimbabwe in lineage III. These Mozambican strains were detected in 2013 in both Manhiça and Mavalane (Supplementary Material 2). Another P[4] strain (RVA/Human-wt/MOZ/0153/2013/G2P[4]), detected in Manhiça, clustered with strains from India and Brazil in a separate sub-cluster in lineage III. The remaining four P[4] Mozambican strains, detected in 2012 in Mavalane, formed a sub-cluster with Southern African strains and a South African porcine strain, in lineage II. The two Mozambican P[8] strains formed a sub-cluster in lineage III with a Ugandan strain and a Belgium strain. Another Belgium strain (RVA/Human-wt/BEL/BE0058/2008/G12P[8]) and a strain detected in Chókwè, Mozambique in 2011 (RVA/Human-wt/MOZ/21196/2011/GXP[8]), clustered separately from the Mozambican strains described in this study (Fig. 3A, Supplementary Material 1). The Mozambican P[6] strains (seven strains detected in 2012) clustered in lineage I. RVA/Human-wt/MOZ/0208/2012/G12P[6] and RVA/Human-wt/MOZ/0286/2012/G12P[6] formed a sub-cluster in lineage I, whereas the remainder of the P[6] strains clustered with a South African strain detected in 2011 (P[6] encoding sequence of mixed infection, RVA/Human-wt/ZAF/2371WC/2008/G9P[8] [24]), a strain from India and a strain detected in Chókwè, Mozambique (Fig. 3A).

All eight Mozambican G12 strains were detected in 2012 and clustered in lineage III (Fig. 3B). Seven of these strains clustered with strains from Chókwè, Mozambique. The remaining G12 strain (RVA/Human-wt/MOZ/0288/2012/G12P[8]) clustered with two Chókwè strains and a Belgian strain. The G12 genotype of strain RVA/Human-wt/MOZ/0288/2012/G12P[8] associated with the P[8] genotype and also clustered with strains in which the G12 genotype was seen to associate with P[8]. The three Mozambican G8P[4] strains, detected in 2012 in Mavalane, clustered in lineage II with a Ugandan and a Zimbabwean strain. Finally, the 12 G2P[4] genotypes, isolated in 2013, clustered in lineage IV. The Mozambican strain (RVA/Human-wt/MOZ/0308/2012/G2P[4]) detected in Mavalane formed a separate sub-cluster, also in lineage IV with a South African strain (RVA/Human-wt/ZAF/MRC-DPRU82/2012/G2P[4]).

### Comparison of VP7 and VP8* antigenic epitopes from Mozambican strains with Rotarix^®^ and RotaTeq^®^

Mozambican VP8* epitopes were compared to Rotarix^®^ (A41CB052A) P[8] and RotaTeq^®^ (WI79-4) P[8] (Table 2). The two Mozambican P[8] strains had identical amino acid profiles and differed only in two of the 25 amino acids in the VP8* epitopes, when compared to both the vaccines. Similarly, all the Mozambican P[6] strains had identical amino acid profiles, although 15 of the 25 amino acids were different from the vaccines. Fourteen of these variations were different to both vaccines while one (position 135) was identical to Rotarix^®^ but different from RotaTeq^®^. The Mozambican P[4] strains did not have identical amino acid profiles; the three G8P[4] strains differed from 11 of the 12 G2P[4] strains at positions 150, 115, 113 and 89. Furthermore, the remaining G2P[4] strain, RVA/Human-wt/MOZ/0308/2012/G2P[4], had an amino acid profile similar to that of the G8P[4] strains, except for a change at position 89, where it was identical to the other G2P[4] strains. Another variation in the G2P[4] group is strain RVA/Human-wt/MOZ/0153/2013/G2P[4], which had a proline at position 114, identical to the P[8] strains and both vaccines, but different from the other G2P[4] strains.

**Table 2:**
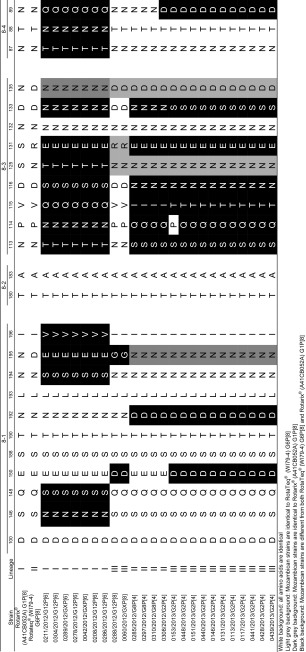
Alignment of the amino acid residues in the VP8* antigenic epitopes of both the Mozambican strains and the rotavirus vaccines

The two VP7 epitopes identified in the Mozambican strains were compared to Rotarix^®^ (A41CB052A) G1 and RotaTeq^®^ (SC2-9) G2 (Table 3). Ten of the 13 G2 Mozambican strains had identical amino acid profiles. Both RVA/Human-wt/MOZ/0428/2013/G2P[4] and RVA/Human-wt/MOZ/0439/2013/G2P[4] had a D146N change which deviates from RotaTeq^®^ (SC2-9) G2. RVA/Human-wt/MOZ/0308/2012/G2P[4] had a S242N change which is identical to the RotaTeq^®^ (SC2-9) G2 profile. The three G8 Mozambican strains had identical amino acid profiles and differed in 12 of the 29 amino acids, when compared with both the vaccines (Table 3). The eight Mozambican G12 strains also had identical amino acid profiles apart from one strain, RVA/Human-wt/MOZ/0288/2012/G12P[8] which had a N130D change. The aspartic acid is identical to the Rotarix^®^ (A41CB052A) G1 strain, whereas the asparagine is identical to the RotaTeq^®^ (SC2-9) G2 strain. Thirteen of the 29 amino acids were different between the G12 Mozambican strains and the vaccines.

**Table 3:**
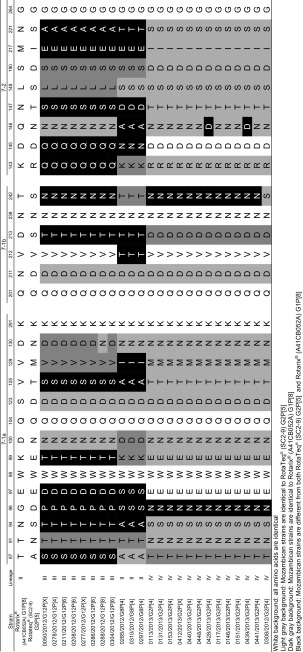
Alignment of the amino acid residues in the VP7 antigenic epitopes of both the Mozambican strains and the rotavirus vaccines

## Discussion

The present study reports the genotyping and molecular characterisation of rotavirus strains from southern Mozambique during 2012-2013. Globally, the most common G and P rotavirus strains are G1-4, G9 and G12 as well as P[4], P[6] and P[8] [25]. In our study, G2P[4] was the prevalent genotype, accounting for 42.3% of strains detected in 2013. This differed from RVA genotypes detected in 2011 in Chókwè, a site situated closely to the area reported in this study. The G2P[4] genotype has been reported worldwide [26] and was also reported to be the predominant type (47.0%) circulating in South African in 2013 [27]. However, it is important to highlight that Mozambique had not yet introduced rotavirus vaccines during the study period while South Africa introduced Rotarix^®^ into their Expanded Programme on Immunisation in 2009.

The second most frequent genotype was G12P[6] (28.8%). The G12 genotype was also detected at lower frequencies with P[8] and P[4] types. Previously, G12P[8] strains were predominant (57.0%) in Mozambique in 2011 [13]. The African Rotavirus Surveillance Network also reported the detection of G12P[6] and G12P[8] in 2012, but at much lower frequencies of 6.0% and 12.0%, respectively [26].

We also detected uncommon genotypes such as G8P[4] and G8P[8], albeit at low rates (4-5.0%). The G8 genotype is known to cause infection in cattle and has been responsible for rotavirus infection in humans, mainly in African countries [28, 29]. Recently it was reported that G8P[6] was the predominant type in São Tome Principe in 2011, being responsible for 71.1% of rotavirus cases [30]. Other genotypes detected in low frequencies were G1P[8] and G9P[8]. Genotype G1P[8] strains were the second most common genotype detected in the Chókwè study [13]. The G9 genotype was not observed at high frequencies in either the current study or the study carried out in Chókwè.

In our study, we observed a difference in distribution of rotavirus strains between the two years. In 2012, G12P[6] was predominant and more rotavirus strain diversity was noted. Genotype G2P[4] was prevalent in 2013 and in less diversity genotypes was observed compared to 2012. Variation in circulating rotavirus genotypes can occur yearly [26], with the change due to the natural variability of rotavirus strains over time. It has been suggested that rotavirus vaccines may influence the distribution of genotypes [31]. A study carried out in Belgium reported a higher prevalence of G2P[4] in vaccinated rotavirus gastroenteritis patients than in unvaccinated rotavirus gastroenteritis patients [32]. In Mozambique, the prevalence of G2P[4] will require continuous monitoring, especially because of the introduction of rotavirus vaccines in 2015.

Geographical differences in genotype distribution within the same country or region can occur [25]. We also observed differences in the geographical distribution of genotypes. In Mavalane, an urban area, G12P[6] (28.6%) was the most prevalent, while in contrast to Manhiça, a rural area, G2P[4] (39.4%) was predominant.

During the study 10.8% (17/157) of the samples were untypeable for G or P, which is comparable to the rate of untypeable samples previously reported from Sub-Saharan countries (8.6-14.6%) [26]. It was noted that in Manhiça a higher number of untypeable strains (16.7%) was observed, when compared to Mavalane, the urban area (6.6%).

No correlation between age group and genotype was observed in this study, although it has been reported previously [33]. In a study investigating the prevalence and genotype distribution of RVA among children with acute gastroenteritis in Kunming, China, genotype G2P[4] was observed in patients between 12 and 48 months of age [34]. However, here we observed the occurrence of G2P[4] below 12 months of age, and even below 5 months (Fig. 2B).

Analyses of primer binding sites did not shed light on the discrepancies between PCR-genotyping results and nucleotide sequencing. In our study, mixed genotypes were observed in 11.7% (13/111) of the samples typed for G and P. This is similar to the 12-14% mixed genotypes reported by the African Rotavirus Surveillance Network [26].

The phylogenetic analyses provided some evidence on the widespread circulation of rotavirus strains in Mozambique, with similar strains detected in Manhiça, Mavalane and Chókwè (Supplementary Material 1). The Chókwè G12 and P[6] strains phylogenetically clustered with strains detected in the current study indicating that these strains circulated in southern Mozambique from 2011 to 2013 (Fig. 3A, B). Interestingly, no G8 strains were identified in the Chókwè study during 2011 [13].

The genotypes of the Mozambican strains clustered mostly with those of strains detected in other African countries, specifically South Africa (G12, G2, P[6]), Uganda (P[8]), Zambia (G12), and Zimbabwe (P[4]; Fig. 3A, B). South Africa, Zambia and Zimbabwe share borders with Mozambique and the circulation of similar rotavirus strains between these countries is not unusual. The South African strains (RVA/Human-wt/ZAF/MRC-DPRU4090/2011/G12P[6] and RVA/Human-wt/ZAF/MRC-DPRU82/2012/G2P[4]) are closely related to the strains from southern Mozambique (Fig. 3A, B). Strains from other countries, which also grouped with Mozambican strains, were from India (P[4], P[6]), Mauritius (G12) and the Philippines (P[4]; Fig. 3A, B).

One exception, where the Mozambican strains did not cluster close together, is RVA/Human-wt/MOZ/0153/2013/G2P[4]. This strain clustered with an Indian (RVA/Human-wt/IND/mcs65/2011/G8P[4]) and a Brazilian (RVA/Human-wt/BRA/QUI-36-F1/2008/G2P[4]) strain in the P[4] lineage III. However, RVA/Human-wt/MOZ/0153/2013/G2P[4] grouped with the other Mozambican strains in lineage G2 IV. The sequencing of the VP8* encoding gene suggests that RVA/Human-wt/MOZ/0153/2013/G2P[4] is a reassortant strain. Interestingly, analysis of the VP8* epitope showed that the Mozambican P[4] strains in lineage III had identical amino acid profiles apart from a proline (instead of a glycine) in strain RVA/Human-wt/MOZ/0153/2013/G2P[4] at position 114. The Indian and Brazilian strains that grouped with this Mozambican strain also have a proline at position 114.

Strain RVA/Human-wt/MOZ/0308/2012/G2P[4] is of particular interest. This strain was detected in 2012 in Mavalane and clustered with G8P[4] Mozambican strains and two South African strains (RVA/Human-wt/ZAF/MRC-DPRU82/2012/G2P[4] and RVA/Pig-wt/ZAF/MRC-DPRU1533/2007/G2P[4]) in P[4] lineage II. However, in the nucleotide tree of segment 9, RVA/Human-wt/MOZ/0308/2012/G2P[4] clustered with the same South African strain (RVA/Human-wt/ZAF/MRC-DPRU82/2012/G2P[4]), separate from the G2 Mozambican strains that circulated in 2013. This grouping of the G2P[4] strain with G8P[4] strains in lineage II of P[4] was previously reported and might indicate a reassortment event or recombination in the VP4-encoding genome segment [35]. RVA/Human-wt/MOZ/0308/2012/G2P[4] also had a VP8* amino acid profile similar to G8P[4] strains but shared a D89 mutation with the other G2P[4] strains.

Lastly, RVA/Human-wt/MOZ/0288/2012/G12P[8] clustered separately from the other G12 Mozambican strains described in this study. However, this strain clustered with two strains detected in Chókwè, Mozambique in 2011 [13]. In the VP7 epitope analysis, RVA/Human-wt/MOZ/0288/2012/G12P[8] was seen to have an identical amino acid profile to the other G12 strains except for one change, N130D. Segment four of this strain grouped with the other Mozambican P[8] strain and had an identical amino acid profile. Therefore RVA/Human-wt/MOZ/0288/2012/G12P[8] is also a possible reassortant strain. However, confirmation of reassortment would have to be performed by whole genome sequencing of these viruses [24, 35–37].

As expected, epitope analysis indicated that Mozambican strains with the same genotype as the rotavirus vaccines shared common amino acids. The VP8* amino acid profiles seemed to be more conserved when compared to VP7 epitopes. Multiple amino acid differences were detected between the vaccine and Mozambican strains of varying genotypes. It is, however, impossible to speculate whether these differences influence antibody attachment and overall protection without specific binding studies being performed.

Many of the amino acids variations detected in the Mozambican strains were also seen in Russian and Belgium strains [23, 24], indicating that these strains are not localized. Belgium G12P[8] strains (RVA/Human-wt/BEL/BE0258/2008/G12P[8] and RVA/Human-wt/BEL/BE0085/2008/G12P[8]) also had a N130D change in the VP7 epitope (7-1a), similar to RVA/Human-wt/MOZ/0288/2012/G12P[8] [38]. These three strains also clustered together with strains from Chókwè in the G12 lineage III (Fig. 3B). The Belgium strains also had a proline at position 96, similar to the Mozambican strains.

Strains isolated from Russia (RVA/Human-wt/RUS/NN2406/2011/G2P[4] and RVA/Human-wt/RUS/NN2578/2011/G2P[4]) had the same S242N change in the VP7 epitope (7-1b) as RVA/Human-wt/MOZ/0308/2012/G2P[4], which is also similar to RotaTeq^®^ (SC2-9) G2 [39]. These strains formed a sub-cluster in lineage IV, separate from other G2P[4] strains (Fig. 3B). RVA/Human-wt/BEL/BE1141/2009/G2P[4] also clustered with RVA/Human-wt/MOZ/0308/2012/G2P[4] and the Russian strains which had the S242N change. RVA/Human-wt/BEL/BE1058/2008/G2P[4], however, clustered with the remaining G2P[4] Mozambican strains with N242 in the 7-1b epitope.

Interestingly, the D146N substitution in the VP7 epitope of the G2P[4] strains was not reflected in the phylogenetic analyses. RVA/Human-wt/MOZ/0439/2013/G2P[4] and RVA/Human-wt/MOZ/0428/2013/G2P[4] clustered with other G2P[4] strains during both phylogenetic analyses, although this isolate had an aspartic acid instead of a asparagine at position 146. The negatively charged aspartic acid at position 146 of the VP7 7-2 epitope in the two Mozambican strains (RVA/Human-wt/MOZ/0428/2013/G2P[4] and RVA/Human-wt/MOZ/0439/2013/G2P[4]) is, however, not seen in the Belgium or Russian strains [23, 24].

The current study, carried out during 2012 and 2013, provides valuable insight into the circulating rotavirus genotypes and strain diversity in southern Mozambique prior to vaccine introduction. The study showed that G2P[4] was prevalent although the study was not carried out for a full two years, which can bias the temporal distribution of genotypes. Phylogenetic and epitope analysis indicated that the Mozambican strains were related with a few potential reassortment events noted. There is evidence for transmission between countries in southern Africa with common strains circulating in southern Mozambique as well as South Africa, Zambia and Zimbabwe.

## Electronic supplementary material

Below is the link to the electronic supplementary material.

Supplementary material 1 (DOCX 213 kb)

Supplementary material 2 (DOCX 14 kb)

Supplementary material 3 (DOCX 296 kb)
