# Supplementary material for: Rotavirus A strains obtained from children with acute gastroenteritis in Mozambique, 2012-2013: G and P genotypes and phylogenetic analysis of VP7 and partial VP4 genes
Source: Arch Virol. 2017 Oct 20;163(1):153–65. doi: 10.1007/s00705-017-3575-y (PMC5756281; doi:10.1007/s00705-017-3575-y)
Supplement: Supplementary file 1 — Supplementary material 1 (DOCX 213 kb) [file 705_2017_3575_MOESM1_ESM.docx]

**Supplementary material 1. Map indicating the sampling sites relative to each other and other southern and eastern African countries.** **A**: Southern and Eastern Africa with Mozambique highlighted; **B**: Part of southern Mozambique with study sites (Manhiça and Mavalane) highlighted. Chókwè, in the Gaza province, where a previous study by Langa and co-workers were carried out [13], is also indicated. The distance between Manhiça and Mavalane is 81 km.


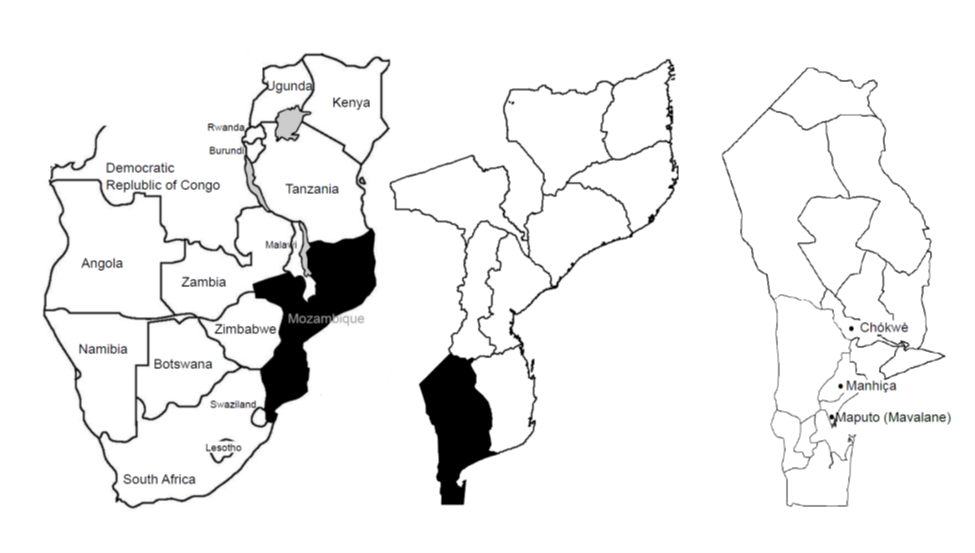

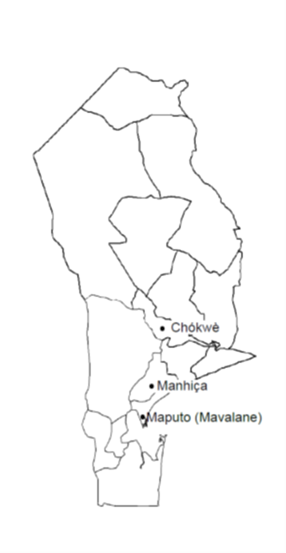


A B
