# Supplementary material for: Rotavirus A strains obtained from children with acute gastroenteritis in Mozambique, 2012-2013: G and P genotypes and phylogenetic analysis of VP7 and partial VP4 genes
Source: Arch Virol. 2017 Oct 20;163(1):153–65. doi: 10.1007/s00705-017-3575-y (PMC5756281; doi:10.1007/s00705-017-3575-y)
Supplement: Supplementary file 2 — Supplementary material 2 (DOCX 14 kb) [file 705_2017_3575_MOESM2_ESM.docx]

| Supplementary material 2: Comparison of G and P typing results using the genotyping PCR and Sanger sequencing | | | | | |
| --- | --- | --- | --- | --- | --- |
|  | **Sample** | **Date** | **Area** | **Genotyping PCR** | **Sanger sequencing** |
|  |  |  |  |  |  |
| 1 | 0042 | 2012 | Manhiça | GXP[8] | GXP[6] |
| 2 | 0050 | 2012 | Manhiça | G12P[X] | G12P[X] |
| 3 | 0060 | 2012 | Manhiça | G12P[X] | GXP[8] |
| 4 | 0113 | 2013 | Manhiça | GXP[4] | G2P[4] |
| 5 | 0117 | 2013 | Manhiça | GXP[4] | G2P[4] |
| 6 | 0131 | 2013 | Manhiça | G2P[4] | G2P[4] |
| 7 | 0146 | 2013 | Manhiça | G2P[4] | G2P[4] |
| 8 | 0151 | 2013 | Manhiça | GXP[4] | G2P[4] |
| 9 | 0153 | 2013 | Manhiça | GXP[4] | G2P[4] |
| 10 | 0208 | 2012 | Mavalane | G12P[6] | G12P[6] |
| 11 | 0211 | 2012 | Mavalane | G12P[6] | G12P[6] |
| 12 | 0277 | 2012 | Mavalane | G12P[6] | G12P[X] |
| 13 | 0278 | 2012 | Mavalane | G12P[6] | G12P[6] |
| 14 | 0285 | 2012 | Mavalane | G8P[4] | G8P[4] |
| 15 | 0286 | 2012 | Mavalane | G12P[6] | G12P[6] |
| 16 | 0288 | 2012 | Mavalane | G12P[8] | G12P[8] |
| 17 | 0289 | 2012 | Mavalane | G12P[6] | GXP[6] |
| 18 | 0297 | 2012 | Mavalane | G8P[4] | G8P[4] |
| 19 | 0304 | 2012 | Mavalane | G12P[6] | G12P[6] |
| 20 | 0308 | 2012 | Mavalane | G2P[4] | G2P[4] |
| 21 | 0310 | 2012 | Mavalane | G8P[4] | G8P[4] |
| 22 | 0412 | 2013 | Mavalane | NT | G2P[X] |
| 23 | 0428 | 2013 | Mavalane | G2P[4] | G2P[4] |
| 24 | 0439 | 2013 | Mavalane | G2P[4] | G2P[4] |
| 25 | 0440 | 2013 | Mavalane | G2P[4] | G2P[4] |
| 26 | 0441 | 2013 | Mavalane | G2P[X] | G2P[4] |
| 27 | 0448 | 2013 | Mavalane | G2P[4] | G2P[4] |

NT: Not typed

X: Unknown
