# Supplementary material for: Rotavirus A strains obtained from children with acute gastroenteritis in Mozambique, 2012-2013: G and P genotypes and phylogenetic analysis of VP7 and partial VP4 genes
Source: Arch Virol. 2017 Oct 20;163(1):153–65. doi: 10.1007/s00705-017-3575-y (PMC5756281; doi:10.1007/s00705-017-3575-y)
Supplement: Supplementary file 3 — Supplementary material 3 (DOCX 296 kb) [file 705_2017_3575_MOESM3_ESM.docx]

A


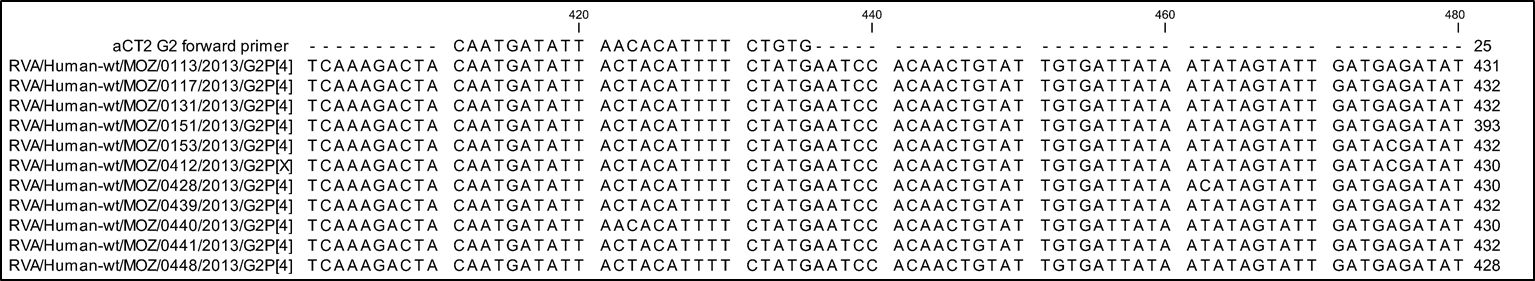


B


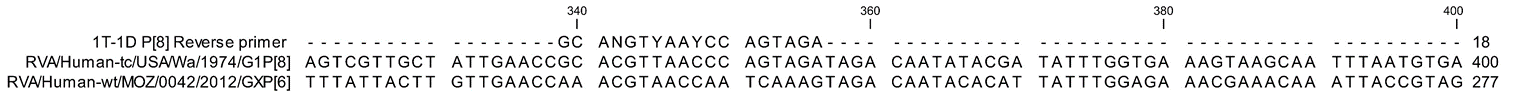


C


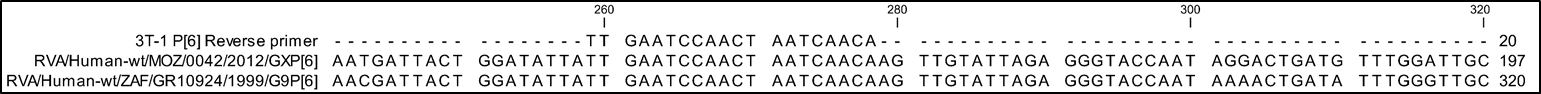


Supplementary material 3. Comparison of binding sites of genotyping primers to nucleotide sequences of Mozambican strains. **A**: The VP7 encoding sequence of RVA/Human-wt/MOZ/0113/2013/G2P[4], RVA/Human-wt/MOZ/0117/2013/G2P[4], RVA/Human-wt/MOZ/0151/2013/G2P[4], RVA/Human-wt/MOZ/0153/2013/G2P[4] and RVA/Human-wt/MOZ/0412/2013/G2P[4] could only be only identified with nucleotide sequencing. The majority of Mozambican strains were similar to the primer sequence of aCT2 apart from position 422, 423 and 433. RVA/Human-wt/MOZ/0440/2013/G2P[4] were identical to the primer sequence apart from position 433. **B**: Alignment of the 1T-1D primer (P[8]) with RVA/Human-tc/USA/Wa/1974/G1P[8] and RVA/Human-wt/MOZ/0042/2012/GXP[6]. **C**: Alignment of reverse primer 3T-1 (P[6]) to RVA/Human-wt/ZAF/GR10924/1999/G9P[6] and strain RVA/Human-wt/MOZ/0042/2012/GXP[6] showed perfect conservation of the primer binding region.
